# Physical activity throughout adolescence and body composition at 18 years: 1993 Pelotas (Brazil) birth cohort study

**DOI:** 10.1186/s12966-016-0430-6

**Published:** 2016-10-01

**Authors:** Virgílio Viana Ramires, Samuel Carvalho Dumith, Fernando Cesar Wehrmeister, Pedro Curi Hallal, Ana Maria Baptista Menezes, Helen Gonçalves

**Affiliations:** 1 Graduate Program in Epidemiology, Federal University of Pelotas, Marechal Deodoro, 1160 - 3 Piso, 96020-220 Pelotas, Rio Grande do Sul Brazil; 2 Graduate Program in Public Health, Federal University of Rio Grande, Rio Grande, Brazil

**Keywords:** Adolescents, Motor activity, Lean mass, Fat mass, Longitudinal studies

## Abstract

**Background:**

Adolescence is a period of accelerated development and increases in body composition. Physical activity (PA) practice has been associated with the development of major components of body composition (bone, muscle and fat). However, the longitudinal effects of PA of different intensities during adolescence are still not well understood. Thus, the main goal this study has investigate the association between practice of moderate- and vigorous-intensity physical activity throughout adolescence and body composition, specifically lean mass (LM) and fat mass (FM), at age 18.

**Methods:**

In this cohort study, physical activity was measured at 11, 15 and 18 years, using questionnaires. Thresholds of 300, 150 and 75 min per week, were used for MVPA, moderate- and vigorous-intensity physical activity, respectively. Consistent physical activity was defined as reaching the thresholds at the three follow-ups. FM and LM at age 18 were assessed by DXA and expressed as fat mass (FMI) and lean mass (LMI) indexes. To verify the association between the trajectories of MVPA, moderate- and vigorous-intensity physical activity in adolescence and FM and LM at 18, multivariate analyses were performed through multiple linear regressions adjusted for co-variables.

**Results:**

A total of 3,176 adolescents were evaluated. The consistent practice of moderate- and vigorous-intensity physical activity according to thresholds during adolescence were directly related to the LMI in boys (moderate-intensity - β = 0.40 and CI95 % 0.13; 0.68 and vigorous-intensity - β = 0.95 and CI95 % 0.69; 1.21) and girls (Moderate-intensity - β = 0.23 and CI95 % 0.02; 0.45 and vigorous-intensity - β = 0.80 and CI95 % 0.29; 1.32). Practice of vigorous-intensity physical activity alone showed to be inversely associated with the FMI in boys (β = -0.53 and CI95 % -0.96;–0.10).

**Conclusion:**

Consistent physical activity practice during adolescence was associated with greater lean mass in both sexes. In boys, vigorous-intensity physical activity was associated with less fat mass.

## Background

Adolescence is characterized as a period of rapid body growth with important changes in body composition [1]. It is also when the body composition standards that tend to remain for life are established [2], which can reflect on the development of morbidities during adulthood [3]. Physical activity practice through adolescence has been associated with benefits in the development of bones [4], muscles [5], and body fat [6]. However, this period has also shown a decrease in the practice of physical activities [7].

The relationships between physical activity practice and adolescent’s body composition have been widely studied. Nevertheless, fat mass (FM) and bone mass (BM) have received more attention [8, 9]. Less is known about the impact of physical activity on lean mass (LM). The few studies that aimed to investigate the relation between physical activity and LM in adolescents report a direct effect between the variables [5, 10, 11]. However, none of these studies isolated the effects of moderate- and vigorous-intensity physical activity. Moreover, only one used a longitudinal study design [6]. Hence, there is no information on the benefits of different physical activities intensities during adolescence regarding LM in early adulthood, particularly with longitudinal data.

The association between physical activity and body fat (BF) has been widely studied since exercise is often recommended as an alternative to reduce weight and BF [12]. Recent findings with adolescents have indicated that physical activity is inversely associated with BF levels, especially moderate and vigorous exercise [13, 14]. Nonetheless, many of these studies employed a cross-sectional design and did not isolate the effects of moderate- and vigorous-intensity physical activities. Therefore, there is still little information regarding the specific effects of moderate- and vigorous-intensity physical activities on BF [15, 16].

The present study aimed to investigate the association among consistent physical activity practice of moderate to vigorous intensity (MVPA), as well as, specifically the moderate- and vigorous intensity during adolescence (between 11 and 18 years old) on FM and LM at 18 years.

## Methods

### Sample

Pelotas is located in the extreme South of Brazil, with approximately 330.000 inhabitants. From January 1st up to December 31st, in the 1993 calendar year, all live births from mothers living at the urban area of the municipality were invited to participate in the birth cohort study. During childhood and adolescence, the 5.249 members of the birth cohort were followed several times. In the adolescence period, they were followed at 11, 15 and 18, with follow-up rates of 87.5 %, 85.7 % and 81.3 %, respectively. Those who showed physical activity data for all three follow-ups in adolescence and body composition (FM and LM) at 18 years old, in addition to the co-variables used to adjust the analyzes were considered with valid data for this study. Detailed information on the cohort can be found in previous papers [17, 18]. The variables used in the analysis of the present study are specified below.

### Body composition variables at 18 years old - outcome

At 18 years old, standing height was measured with a wood and aluminum stadiometer with 0.1 cm precision. The weight was measured using a precision scale (0.01 kg). The FM and LM levels were measured through dual-energy x-ray absorptiometry using a DXA device (Lunar Prodigy, GE Healthcare, USA). This exam was performed with the adolescents in supine position and all metal accessories (earrings, body piercings, rings, etc.) were removed. Adolescents with metal orthopedic devices or who were pregnant did not undergo this exam. In the body composition measurements, the subjects wore light, tight spandex clothes (shorts and sleeveless shirt) and no shoes. All procedures were performed by a team previously trained to operate all equipment and carry out anthropometry.

Given the direct relation between FM and LM with the subjects’ height, the authors chose to express these variables as fat-mass index (FMI) and lean-mass index (LMI) [19]. These indices were generated from the FM and LM readings from the DXA device. Since the total body weight informed by the DXA device comprises the sum of the fat, lean, and bone masses and since this weight does not equal the total body mass – measured with a precision scale –, FM and LM were adjusted to the total weight according to the following equations:

$$ \mathrm{LMadjusted}=\frac{\mathrm{LM}}{{\mathrm{TBM}}_{\mathrm{DXA}}}x\ {\mathrm{TBM}}_{\mathrm{M}}\kern0.5em \mathrm{and}\ \mathrm{FMadjusted}=\frac{\mathrm{FM}}{{\mathrm{TBM}}_{\mathrm{DXA}}}x\ {\mathrm{TBM}}_{\mathrm{M}},\ \mathrm{where} $$

FM = fat mass (kg); LM = lean mass (kg); TBM_DXA_ = total body mass (kg), the sum of fat, lean, and bone masses (kg) provided by the DXA device; and TBM_M_ = total body mass (kg) measured.

After this procedure, the FM and LM variables were adjusted to the subjects’ height with their respective indices according to the equations:

$$ \mathrm{F}\mathrm{M}\mathrm{I}=\frac{\mathrm{FMadjusted}}{{\mathrm{H}}^2}\ \mathrm{e}\ \mathrm{L}\mathrm{M}\mathrm{I} = \frac{\mathrm{LMadjusted}}{{\mathrm{H}}^2},\ \mathrm{where} $$

FMI = fat mass index (kg/m^2^); LMI = lean mass index (kg/m^2^); and H = height (m).

### Physical activity variables - exposition

Physical activity practice at 11, 15, and 18 years old was measured through questionnaires. At 11 and 15, the subjects were asked about how they commuted to school and about their leisure physical activities from a list of 13 activities (soccer, indoor soccer, track and field, basketball, dance, gymnastics, martial arts, swimming, volleyball, tennis/paddle, handball, dodgeball, and street cricket). The subjects could also report three activities not in the list. To each activity in the list, adolescents were asked if they did the activity and in case of a positive response, they were asked about the frequency of participation in these activities, in days per week, and then an average duration in minutes per participation. In order to adapt the instrument to the age group and local context, at 15, the modalities handball, dodgeball, and street cricket were replaced by hiking, weight lifting, and gym. The questionnaires may be accessed in (www.epidemio-ufpel.org.br). At 18, physical activity at leisure and commuting was inquired using the long version of the International Physical Activity Questionnaire (IPAQ) (www.ipaq.ki.se) [20].

The moderate- and vigorous physical activity score in each follow-up was tallied based on the sum of the time spent with leisure and commuting physical activity as follow.

From this list of leisure sports activities and the questions pertaining to the commute to school in the instruments used at 11 and 15 years old, the activities were classified as moderate and vigorous while observing the thresholds for physical activity intensity in metabolic equivalents (METs) – a unit used to estimate the metabolic cost of physical activities (<3.0 METs = light; 3.0 − 6.0 METs = moderate, and >6.0 METs = vigorous), proposed by Pate et al. (1995) [21]. Based on the physical activity compendium for adolescents created by Ridley et al. (2008) [22], the mean of three possible MET amounts spent for each modality was calculated while considering the possibility of light, moderate, and vigorous intensities. After the mean MET values were obtained for each modality in the instrument, they were classified as light, moderate, or vigorous-intensity. For example: According to the compendium for adolescents, handball can be practiced according to the following intensities, in METs: light = 6.0 METs, moderate = 8.0 METs, and vigorous = 10 METs. In this case, the average in METs of handball is 8.0 METs, thus it is classified as a vigorous-intensity activity.

The same procedure applied to the leisure activities was used to classify commuting, hiking, and cycling. For weight lifting, practiced at 15 years old, although it is a light-intensity activity [22], the authors chose to consider its energy expenditure in METs according to the compendium created for adults, hence it was classified as a moderate-intensity activity [23]. The reason for this choice is that, in the local context, this activity is usually practiced in gyms with moderate- and vigorous- intensity to develop muscle mass or achieve weight loss. At 18 years old, the leisure and commuting physical activities were classified and grouped into moderate- and vigorous-intensity according to IPAQ’s guidelines for processing and analysis [20].

After each activity have been classified according their intensity, variables of MVPA, moderate- and vigorous-intensity physical activity were created in accordance to the time spend in minutes per week. The MVPA score was tallied based on the sum of the time spent with moderate- and vigorous physical activity.

In order to verify the relation between time practicing moderate- and vigorous-intensity physical activity at each age (11, 15, and 18 years old) and FM and LM at 18, time tertiles were used for each intensity. For the main analysis, trajectories of MVPA, moderate and vigorous intensity physical activity were created based on weekly minutes spent on each intensity at 11, 15 and 18 years of age, as follows. Initially, the variables were dichotomized according to the time thresholds equal to or greater than 300, 150, and 75 min of weekly MVPA, moderate- and vigorous-intensity physical activity practice, respectively. The threshold to MVPA (300 min/w) was defined according to the recommendations. Thresholds for moderate and vigorous intensity physical activity in adolescents were not found. Thus, thresholds to moderate (150 min/w) and vigorous (75 min/w) intensity physical activity used in this study were defined based on a minimal energy expenditure of 13 MET-hours per week which is equivalent to walking 150 min per week at a 4 mile per hour pace or 75 min per week at a 6 mile per hour pace, representing moderate and vigorous intensity, respectively. The times and intensities mentioned above show a relationship with body composition parameters [24].

After dichotomization, variables were generated to describe the trajectories according to the thresholds above: *has never reached*, *reached once*, *reached twice*, and *has always reached*. In addition, time quartiles of moderate- and vigorous intensity physical activity accumulated in the three follow-up periods were built from the sum of the minutes of moderate and vigorous-intensity physical activity. The reason to choose the variable of physical activity accumulated in quartiles was because it allows better view of the higher and lower groups of times of practice and dose-response effect.

### Co-variables

A series of variables of the adolescents and their mothers that are independently related to physical activity practice or to FM and LM were selected to adjust the analyses for potential confounding factors. The variables from the adolescents were: Socioeconomic level, measured through the asset index (IEN) [25], expressed in quintiles; self-reported skin color (white, black, *pardo* [official term used in the Brazilian census, meaning dark-skinned], yellow, and Indian); continuous body mass index (BMI); screen time (TV, videogame, and computer) in hours per day; use of medicine for weight-loss (yes or no); diet to weight-loss prescribed by physician or nutritionists (yes or no); high fat intake, obtained through the Block Fat Screener [26], expressed in high or low fat intake; all collected at 11 years old. In addition, sexual maturity stage of adolescents was self-reported at 15 through the Tanner scale for pubic hair [27]. The mother’s variables were: schooling, in years of education completed; physical activity, in minutes per week (active ≥ 150 min/week); and continuous BMI, all collected at the 11-year-old follow-up.

### Statistical analyses

The main characteristics of the sample are described as means and standard deviations for the continuous variables and as absolute and relative frequencies for the categorical variables. Bivariate analyses were performed by applying simple linear regressions to verify the raw association between the moderate- and vigorous-intensity physical activity variables, as well as the trajectories of MVPA, moderate- and vigorous-intensity physical activity with FM and LM at 18 years old. In order to verify the association between the trajectories of MVPA, moderate- and vigorous-intensity physical activity in adolescence and FM and LM at 18, multivariate analyses were performed by applying multiple linear regressions adjusted for co-variables. Analyses stratified by sex were performed by considering the sexual dimorphism in the development of FM and LM [1, 28]. The results of the regressions are presented as regression coefficients (**β**) and their respective confidence intervals of 95 % (CI95 %). Values of *p* below 0.05 for differences between means or for linear trends were accepted as statistically significant. All analyses were carried out using the statistical package Stata version 12.1.

## Results

The final sample analyzed in the present study was made up of 3.176 adolescents who had valid data for the exposition and outcome variables beyond the co-variables. Of these 1.637 (51.5 %) were female.

The demographics, physical activity, screen time, maturity and body composition data of adolescents are described in Table 1.

**Table 1:** Characteristics of the sample. The 1993 Pelotas (Brasil) Birth Cohort

Variables	Boys	Girls
	n(%)	n(%)
Skin color		
Black	1480 (68.2)	1473 (65.5)
White	271 (12.6)	284 (12.6)
Brown	316 (14.5)	388 (17.2)
Yellow	44 (2.0)	49 (2.2)
Indigenous	58 (2.7)	57 (2.5)
Asset índex in quintiles		
1st (poorest)	445 (21.1)	418 (19.2)
2nd	414 (19.6)	437 (20.1)
3rd	401 (19.0)	456 (21.0)
4rd	414 (19.6)	443 (20.4)
5rd (richest)	436 (20.7)	420 (19.3)
Tanner stage (pubic hair)		
1	9 (0.5)	56 (2.7)
2	85 (4.7)	238 (11.4)
3	374 (20.5)	811 (38.8)
4	774 (42.3)	784 (37.5)
5	586 (32.0)	200 (9.6)
MVPA 11, 15 and 18y (≥300 min/week)		
Has never reached	112 (6.3)	474 (24.6)
Reached once	391 (21.9)	741 (38.5)
Reached twice	725 (40.5)	544 (28.3)
Has always reached	559 (31.3)	164 (8.6)
Physical activity (moderate-intensity)11, 15 and 18y (>150 min/week)		
Has never reached	190 (10.5)	219 (11.3)
Reached once	588 (32.4)	555 (28.5)
Reached twice	702 (38.6)	712 (36.5)
Has always reached	337 (18.5)	462 (23.7)
Physical activity (vigorous-intensity) 11, 15 and 18y (>75 min/week)		
Has never reached	184 (10.2)	1.095 (56.2)
Reached once	436 (24.1)	657 (33.7)
Reached twice	696 (38.4)	173 (8.9)
Has always reached	495 (27.3)	24 (1.2)
Screen time at 11y (hours/day) in tertiles - Mean (SD)		
Upper	7.7 (2.2)	7.5 (1.9)
Middle	4.3 (0.5)	4.3 (0.6)
Bottom	1.9 (0.9)	1.9 (0.9)
Body mass index at 11y in tertiles - Mean (SD)		
Upper	22.6 (3.0)	22.6 (3.0)
Middle	17.7 (0.8)	17.8 (0.8)
Bottom	15.4 (0.8)	15.2 (1.0)
Fat mass (kg) - Mean (SD)	12.8 (9.4)	21.9 (9.3)
Lean mass (kg) - Mean (SD)	54.3 (6.0)	36.5 (4.4)
Fat mass index at 18y - Mean (SD)	4.2 (3.0)	8.4 (3.6)
Lean mass index at 18y - Mean (SD)	18.0 (1.6)	13.2 (1.4)

*MVPA* Moderate to vigorous physical activity

The distribution of the time spent with MVPA, moderate- and vigorous-intensity physical activity during adolescence is presented in Table 2. The time the boys spent per week with MVPA and vigorous-intensity physical activity was higher than the time spent by girls at 11, 15, and 18 years old. When moderate-intensity physical activity was considered, the girls’ weekly time was higher than the boys’ at 11 and 15, but the boys’ was higher than the girls’ at 18. Also, the percentage of boys who reached or exceeded the recommendations of MVPA practice for adolescents (≥300 min/week) was also higher than among the girls. Furthermore, the data show that both boys and girls increase physical activity during adolescence, especially the MVPA and moderate intensity physical activity in boys and girls and vigorous intensity physical activity in boys.

**Table 2:** MVPA, moderate and vigorous-intensity physical activity measured at 11, 15 and 18 years. The 1993 Pelotas (Brasil) Birth Cohort

Follow-up		% Active (≥300 min/week)		MVPA (min./week)		Moderate-intensity PA (min./week)		Vigorous-intensity PA (min./week)	
		N	%	n	Median (IQR)	n	Median (IQR)	n	Median (IQR)
11 years	Total	4293	48.0	4293	280 (140-545)	4312	150 (75-290)	4311	30 (0-180)
	Male	2120	58.4	2120	370 (180-662)	2130	140 (60-265)	2128	120 (16-330)
	Females	2173	38.0	2173	220 (110-415)	2182	167 (85-315)	2183	0 (0-45)
15 years	Total	4324	48.2	4324	280 (120-580)	4326	120 (50-245)	4326	0 (0-195)
	Male	2110	62.6	2110	420 (190-810)	2115	100 (30-200)	2115	150 (0-420)
	Females	2214	34.5	2214	200 (90-390)	2211	150 (50-280)	2211	0 (0-0)
18 years	Total	4060	57.3	4060	360 (152-680)	4104	240 (120-490)	4099	0 (0-360)
	Male	1989	69.5	1989	480 (250-850)	2014	300 (150-600)	2010	120 (0-600)
	Females	2071	45.6	2071	250 (120-510)	2090	210 (90-420)	2089	0 (0-0)

*MVPA* Moderate to vigorous physical activity, *IQR* Interquartile range

### Association between physical activity and FMI and LMI

Table 3 shows the relations between tertiles of moderate- and vigorous-intensity physical at 11, 15, and 18 years old with FMI and LMI at 18. The raw analyses showed inverse relations between FMI with vigorous-intensity physical activity at 15 years old and moderate-intensity physical activity at 18 for males only. In the analysis of the association between tertiles of moderate- and vigorous-intensity physical activities and LMI, data showed that both are directly related to LMI for boys and girls at all 3 ages of follow-up, except for males at 11 years old (Table 3).

**Table 3:** Association between moderate and vigorous-intensity physical activity at 11, 15 and 18 years with body fat mass and lean mass index at 18 years in boys and girls belonging to the 1993 Pelotas (Brazil) Birth Cohort

Physical Activity Intensity in Tertiles	Fat Mass Index (FMI)						Lean Mass Index (LMI)					
	*N*	Boys		*n*	Girls		*n*	Boys		*N*	Girls	
		β (95 % CI)	*P*		β (95 % CI)	*p*		β (95 % CI)	*p*		β (95 % CI)	*P*
Moderate-intensity physical activity at 11y	1782		0.7	1842		0.3	1782		0.003	1842		0.005
1st	724	0		590	0		724	0		590	0	
2nd	548	-0.14 (-0.48; 0.20)		577	0.00 (-0.42; 0.40)		548	0.13 (-0.04; 0.29)		577	0.11 (-0.06; 0.28)	
3rd	510	-0.01 (-0.36; 0.33)		675	0.27 (-0.13; 0.66)		510	0.30 (0.13; 0.48)		675	0.26 (0.10; 0.43)	
Vigorous-intensity physical activity at 11y	1780		0.4	1843		0.6	1780		0.4	1843		0.008
1st	413	0		1231	0		413	0		1231	0	
2nd	485	-0.07 (-0.48; 0.33)		369	-0.14 (-0.56; 0.27)		485	0.09 (-0.11; 0.29)		369	0.06 (-0.11; 0.23)	
3rd	882	-0.23 (-0.59; 0.13)		243	-0.20 (-0.70; 0.28)		882	0.12 (-0.07; 0.30)		243	0.32 (0.12; 0.52)	
Moderate-intensity physical activity at 15y	1796		0.6	1914		0.2	1796		0.008	1914		<0.001
1st	681	0		553	0		681	0		553	0	
2nd	609	-0.18 (-0.51; 0.16)		631	0.18 (-0.22; 0.59)		609	-0.08 (-0.25; 0.08)		631	0.15 (-0.01; 0.31)	
3rd	506	-0.03 (-0.39; 0.32)		730	0.36 (-0.03; 0.75)		506	0.20 (0.02; 0.38)		730	0.31 (0.15; 0.47)	
Vigorous-intensity physical activity at 15y	1796		0.001	1914		0.2	1796		<0.001	1914		0.02
1st	436	0		1533	0		436	0		1533	0	
2nd	390	-0.50 (-0.92; -0.09)		223	0.07 (-0.43; 0.57)		390	0.28 (0.07; 0.49)		223	0.22 (0.02; 0.42)	
3rd	970	-0.65 (-1.00; -0.30)		158	-0.50 (-1.09; 0.07)		970	0.50 (0.34; 0.68)		158	0.23 (0.00; 0.46)	
Moderate-intensity physical activity at 18y	1901		0.001	1949		0.07	1901		0.03	1949		<0.001
1st	500	0		779	0		500	0		779	0	
2nd	642	-0.08 (-0.43; 0.28)		658	0.27 (-0.09; 0.64)		642	0.12 (-0.06; 0.30)		658	0.27 (0.12; 0.42)	
3rd	759	-0.56 (-0.90; -0.21)		512	0.45 (0.05; 0.84)		759	0.24 (0.06; 0.41)		512	0.50 (0.34; 0.66)	
Vigorous-intensity physical activity at 18y	1899		0.4	1949		0.3	1899		<0.001	1949		<0.001
1st	786	0		1459	0		786	0		1459	0	
2nd	209	0.30 (-0.15; 0.77)		101	0.21 (-0.50; 0.93)		209	0.10 (-0.13; 0.34)		101	0.36 (0.07; 0.65)	
3rd	904	0.10 (-0.19; 0.39)		389	0.29 (-0.11; 0.69)		904	0.46 (0.30; 0.60)		389	0.34 (0.18; 0.50)	

Wald test for heterogeneity

The relations between the MVPA, moderate- and vigorous-intensity physical activity trajectory with FMI and LMI for boys and girls are presented in Table 4. After the adjusted analyses between the physical activity trajectories and FMI, it was found that only the vigorous physical activity trajectory among boys was inversely related to FMI (adjusted *p* value = 0.03). When the relation between the MVPA, moderate- and vigorous-intensity physical activity trajectories during adolescence and LMI was tested, an important direct effect of physical activity on LM of 18-year-old for boys and girls was observed. Among boys, a dose-response effect could be observed for all physical activity trajectories in adjusted analysis. In girls, only the vigorous-intensity physical activity trajectory showed a dose-response effect among the categories analyzed (adjusted *p* value < 0.001).

**Table 4:** Association between physical activity trajectory of MVPA, moderate- and vigorous intensity at 11, 15 and 18 years with body fat and lean mass index at 18 years stratified by sex. The 1993 Pelotas (Brazil) Birth Cohort

Trajectories of physical activity 11, 15 and 18 years		Fat Mass Index (FMI)						Lean Mass Index (LMI)					
		*n*	Crude		*n*	Adjusted†		*n*	Crude		*n*	Adjusted†	
			β coefficient (95 % CI)	*p*		β coefficient (95 % CI)	*p*		β coefficient (95 % CI)	*p*		β coefficient (95 % CI)	*P*
Boys	MVPA (≥300 min/wk)	1703		*0.04	1368		*0.4	1703		^§^ < 0.001	1368		^§^ < 0.001
	Has never reached	113	0		93	0		113	0		93	0	
	Reached once	406	-0.86 (-1.50; -0.22)		323	-0.42 (-0.93; 0.08)		406	0.80 (0.47; 1.14)		323	0.57 (0.27; 0,87)	
	Reached twice	681	-0.79 (-1.40; -0.18)		546	-0.35 (-0.83; 0.13)		681	0.98 (0.66; 1.30)		546	0.83 (0.54; 1.12)	
	Has always reached	503	-0.87 (-1.50; -0.25)		406	-0.26 (-0.75; 0.24)		503	1.13 (0.81; 1.46)		406	0.99 (0.69; 1.29)	
	Moderate-intensity (≥150 min/wk) (>150 min/week)	1731		*0.3	1386		*0.6	1731		^§^ < 0.001	1386		^§^0.004
	Has never reached	180	0		149	0		180	0		149	0	
	Reached once	555	-0.46 (-0.98; 0.06)		437	0.02 (-0.38; 0.43)		555	0.23 (-0.03; 0.49)		437	0.25 (0.00; 0.50)	
	Reached twice	670	-0.47 (-0.97; 0.04)		542	0.05 (-0.35; 0.45)		670	0.29 (0.04; 0.54)		542	0.29 (0.05; 0.53)	
	Has always reached	326	-0.34 (-0.90; 0.22)		258	0.23 (-0.21; 0.68)		326	0.55 (0.27; 0.83)		258	0.40 (0.13; 0.68)	
	Vigorous-intensity (≥75 min/wk)	1727		*0.02	1384		*0.03	1727		^§^ < 0.001	1384		^§^ < 0.001
	Has never reached	174	0		134	0		174	0		134	0	
	Reached once	410	-0.90 (-1.45; -0.36)		323	-0.64 (-1.08; -0.20)		410	0.48 (0.20; 0.75)		323	0.53 (0.27; 0.80)	
	Reached twice	667	-0.86 (-1.36; -0.34)		536	-0.54 (-0.95; -0.13)		667	0.54 (0.28; 0.80)		536	0.62 (0.37; 0.87)	
	Has always reached	476	-0.74 (-1.27; -0.20)		391	-0.53 (-0.96; -0.10)		476	0.88 (0.62; 1.15)		391	0.95 (0.69; 1.21)	
Girls	MVPA (≥300 min/wk)	1797		*0.1	1576		*0.3	1797		* < 0.001	1576		* < 0.001
	Has never reached	467	0		405	0		467	0		405	0	
	Reached once	683	0.47 (0.05; 0.89)		597	0.20 (-0.10; 0.50)		683	0.24 (0.07; 0.41)		597	0.13 (-0.02; 0.28)	
	Reached twice	499	0.45 (0.00; 0.90)		441	0.19 (-0.13; 0.52)		499	0.51 (0.33; 0.70)		441	0.34 (0.18; 0.50)	
	Has always reached	148	0.37 (-0.29; 1.03)		133	-0.13 (-0.61; 0.34)		148	0.49 (0.22; 0.76)		133	0.23 (-0.00; 0.47)	
	Moderate-intensity (≥150 min/wk)	1820		*0.1	1594		*0.9	1820		* < 0.001	1594		*0.006
	Has never reached	205	0		176	0		205	0		176	0	
	Reached once	522	0.30 (-0.27; 0.88)		457	0.02 (-0.39; 0.44)		522	0.21 (-0.02; 0.45)		457	0.16 (-0.04; 0.37)	
	Reached twice	661	0.43 (-0.13; 0.99)		580	0.07 (-0.33; 0.48)		661	0.51 (0.28; 0.74)		580	0.34 (0.13; 0.54)	
	Has always reached	432	0.68 (0.08; 1.28)		381	-0.05 (-0.48; 0.38)		432	0.50 (0.26; 0.74)		381	0.23 (0.02; 0.45)	
	Vigorous-intensity (≥75 min/wk)	1821		*0.7	1595		*0.3	1821		^§^ < 0.001	1595		^§^ < 0.001
	Has never reached	1014	0		873	0		1014	0		873	0	
	Reached once	618	-0.02 (-0.38; 0.33)		549	-0.04 (-0.29; 0.22)		618	0.26 (0.12; 0.40)		549	0.27 (0.14; 0.39)	
	Reached twice	165	-0.32 (-0.91; 0.27)		152	0.04 (-0.37; 0.45)		165	0.28 (0.04; 0.52)		152	0.35 (0.14; 0.56)	
	Has always reached	24	-0.24 (-1.69; 1.21)		21	-0.94 (-1.97; 0.09)		24	1.04 (0.46; 1.63)		21	0.80 (0.29; 1.32)	

*P value of heterogeneity; § *P* value for trend; † Adjusted for: Asset index, skin color, medicine use for weight loss, weight loss diet, fat intake, BMI, screen time (hours/week) and adolescent schooling at 11 years, physical activity mother and mother's BMI, collected at 11 years and sexual maturation (Tanner stages/pubic hair) collected at 15 year

Has never reached, Reached once, Reached twice and Has always reached = times that reach the thresholds of the ≥300 (min/wk), ≥150 (min/wk) and ≥75 (min/wk) for *MVPA*, moderate- and vigorous intensity physical activity, respectively, to 11, 15 and 18 years

**Fig. 1:**
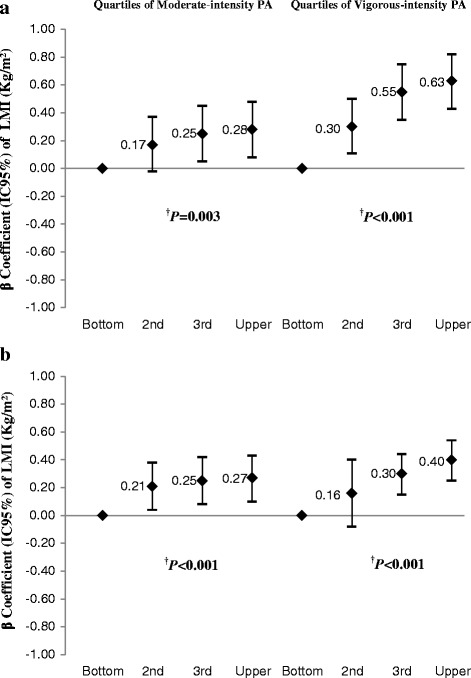
*Adjusted for: Asset index, skin color, medications for weight loss, weight loss diet, fat intake, BMI, screen time (hours/week) and adolescent education, physical activity mother and mother's BMI, collected at 11 years and sexual maturation (Tanner stages/pubic hair) collected at 15 year; †*P* value of tendency; FMI = Fat mass index; LMI = Lean mass index. Effect of moderate- and vigorous-intensity physical activity accumulated at 11, 15 and 18 years on LMI in boys (**a**) and girls (**b**) belonging to the 1993 Pelotas (Brasil) Birth Cohort

The association between quartiles of minutes accumulated moderate- and vigorous-intensity physical activity at 11, 15, and 18 years old with FMI and LMI in boys and girls at 18 are showed in Fig. 1. No effect was observed between the accumulated time of moderate- and vigorous-intensity physical activity and FMI. When LMI was considered, a positive association was found between the accumulated time of moderate- and vigorous-intensity physical activity and LMI at 18 years old, for boys and girls, with a dose-response effect observed in raw and adjusted analyses. The greatest magnitude of the association on the LMI of boys and girls was observed for vigorous physical activity (Fig. 1).

## Discussion

By investigating the longitudinal and cross-sectional association between physical activity practice during adolescence and body composition in early adulthood, particularly FM and LM, the main findings were: (i) physical activity practice increase in both sexes; (ii) boys and girls who practiced physical activity for more time at 11, 15, and 18 years old or who maintained physical activity practice MVPA, moderate- and vigorous-intensity, according to the thresholds established, throughout adolescence had higher LM levels at 18 years old, with a greater effect promoted by vigorous physical activity; (iii) the consistent practice of vigorous physical activity during adolescence resulted in lower FM levels for boys at 18 years old; (iv) the age of 15 was when vigorous-intensity physical activity had the greatest impact on FM of boys at 18.

It is common knowledge that the adolescence period is characterized by relevant decreases in physical activity [7]. On the other hand, the results from this study show an increase in physical activity practice for both sexes. Among boys, the most consistent increased was observed for MVPA, while for girls this increase was found for moderate physical activity. Although the results are surprising for this age group, other studies carried out with adolescents in Pelotas have also found similar results [29, 30]. Regarding the increase in physical activity practice, we believe it may be the result of campaigns developed in the last few years promoting physical activity practice, as well as aspects related to the cult of a well-built body, a behavior which is very prevalent in this age group. As regard to the differences observed in the intensities of the practiced activities, we believe that such effects may be consequence of the types of activities more practiced by this age group in the local context, in which girls are more involved in activities such as walking, gymnastics and dance whereas boys in sports practice such as soccer, basketball and handball.

Studies investigating the relation between physical activity practice and body composition showed that total physical activity, MVPA, moderate - and vigorous-intensity physical activities are directly associated with free fat mass during adolescence [5]. Other studies showed that total physical activity and MVPA are associated with LM throughout adolescence [10, 11]. However, none of these studies assessed the specific associations between moderate- and vigorous-intensity physical activities and LMI.

In the present study, the assessment of the specific effects of moderate- and vigorous-intensity physical activities on LMI at 18 years old showed that boys and girls who practiced physical activity for more time at 11, 15, and 18 had higher LMI at 18 years old, except for vigorous-intensity in boys at 11 years. When the consistent practiced of MVPA, moderate- and vigorous intensity physical activity during adolescence were considered, those who reached the threshold for the referred intensities showed higher LMI levels at 18 years old, with a greater effect promoted by vigorous-intensity physical activity. It is noteworthy that most relevant effects of moderate- and vigorous physical activity practice on lean mass found for girls according to data showed in Table 3 leading to an important confounding, once that is showing crude analysis only. Additionally, in Table 4, after adjusted analysis a higher magnitude was observed for boys.

Adolescence is characterized by rapid growth with important increases in muscle mass as a natural result [1]. Nevertheless, the differences detected in adolescents who had more weekly minutes of physical activity practice and of way more consistently during this period indicate that there are physiological mechanisms in this process that are favored by physical activities, particularly vigorous ones. In this sense, the most likely explanation for the present findings is the greater stimulation of the activity of anabolic hormones such as testosterone, growth hormone, and hormonal growth factors [31, 32] involved in the process of increase and maintenance of LM [33–35].

The inverse effect of physical activity on BF, especially vigorous-intensity physical activity, has been shown in cross-sectional [13] and longitudinal [15] studies. In the analyses of the present study, after adjusting for a series of variables known to impact the development of BF, only the vigorous-intensity physical activities among boys were inversely associated with FM at 18 years old. These findings match two other studies with similar methodologies as this one [36, 37].

The physiological and metabolic adaptations stimulated by physical activity practice comprising a set of mechanisms which allow the reduction of BF through metabolic demands imposed the body [38, 39]. Thus, they are pointed as the most plausible reasons for the present findings. Among other adaptations, these stand out: The higher energy expenditure [39, 40] required by the greater energy demand imposed by vigorous movements [41]; the higher rate of fat oxidation [42, 43], a result of the higher activity of oxidative enzymes [44, 45] and of lipolytic hormones [32, 46]; and the increased resting metabolic rate (RMR) [47, 48] through the increase in oxygen uptake after the practice of vigorous-intensity physical activity [43, 49] and by the greater amount of skeletal muscle tissue [5, 10].

Regarding the differences identified between the sexes, the most likely reasons for not finding an inverse relation between physical activity and BF in girls are: the fact that adolescence is a period of great FM acquisition for girls; the hormonal differences that are unfavorable for females in reducing FM [1]; and the lower amount of vigorous-intensity physical activity among girls throughout adolescence. The lack of sampling power to detect a statistically significant difference in favor of those who practiced vigorous-intensity physical activity for 75 min/week or more at the 3 ages assessed must also be considered. Furthermore, during adolescence, boys have a great capacity of oxidizing fat [50].

It is important to highlight that physical activity and caloric intake are the main elements in the energetic balance and both are important in body composition changes especially in maintaining weight and preventing weight and body fat gain [51]. Experimental studies state that when diet and physical activities are administrated together, they promote stronger effects on body composition [12, 52, 53]. In order to reduce weight and body fat, a negative energetic balance as a result from consumed energy and spent energy is expected. Therefore, if the caloric intake is higher than energetic expenditure, the effects of physical activity may not be observed [54]. In this study, it was not possible to observe the effect of a higher amount of physical activity on body fat in some associations which may be the result of a positive energetic balance. However, it is important to highlight that although we do not have measures for caloric intakes, our analysis were adjusted for diets with high consumption of fat possibly indicating a diet with high calories. Therefore, we believe that the lack of association between physical activity practice and a lower amount of body fat is mainly due to the low amount of vigorous activity and the lack of sample power in some categories.

Some important issues can be considered limitations in the present study. The practice of physical activity at 11, 15, and 18 years old was measured through questionnaires, which allows for classification errors inherent to the instruments such as over or underestimated answers. However, the instrument used at 11 and 15 years old has been used in several follow-ups with participants of Pelotas birth cohorts (1982, 1993 and 2004) [30], also in cross-sectional surveys with adolescents of Pelotas city [29, 55]. Additionally, it shows good reliability and allows a wide description of commuting and leisure time physical activity [55]. At 18 years old, the IPAQ questionnaire was used, an instrument validated by using accelerometers as criterion method and that has been widely used in studies with large samples [56, 57]. The fact that two different instruments were used to measure physical activity at 11 and 15 and at 18 years old can be minimized since only leisure and commuting activities were considered, which were standardized and expressed as MVPA, moderate, and vigorous physical activity with the same thresholds applied to all periods. In addition, the moderate- and vigorous-intensity physical activities were classified based on the specific compendium for adolescents [22] and on the guideline for processing and analyzing IPAQ data [20]. Other condition that could be considered as a limitation of study was the fact that physical activities of light intensity as well as the domestic and occupational were not included as variables. However, the main goal of this study was regarded to the relationships between MVPA, moderate- and vigorous intensities physical activity. Furthermore, the used instruments to measure physical activity are less suitable to measures light, domestics and occupational physical activity. In addition, the lack of sample power in many categories was responsible for the weak or no association found in several analyses.

Among the strengths of the present study, the following aspects stand out: (i) The 3 measurements of physical activity during adolescence representing the ages at the beginning (11 years old), middle (15 years old), and end (18 years old) of this phase allowed assessing the change over the period and to plot trajectories; (ii) using DXA allowed FM and LM to be specifically measured; (iii) the outcomes of FM and LM were adjusted for the height of the subjects by transforming the absolute FM and LM measures into indices (FMI and LMI); (iv) the analyses were adjusted for a series of variables known to affect the development of FM and LM; and (v) being a population cohort study carried out in Brazil.

## Conclusions

In summary, maintaining high levels of physical activity practice during adolescence, particularly vigorous-intensity activity was related to greater LM levels in both sexes and to a lower FM level among 18-year-old boys. Even if physical activity practice did not have direct relationship on FM levels among girls, the effect of vigorous-intensity physical activity on LM gain suggests that this may be an important strategy to increase energy expenditure and RMR, besides aiding in maintaining or reducing BF.

Thus, the results suggest that accumulating 75 min/week or more of vigorous-intensity physical activity throughout adolescence seems appropriate to promote changes in body composition of boys and girls and, hence, is able to aid in fighting the overweight and obesity epidemic.
